# Spatiotemporal heterogeneity of land subsidence in Beijing

**DOI:** 10.1038/s41598-022-16674-6

**Published:** 2022-09-06

**Authors:** Guangyao Duan, Huili Gong, Beibei Chen, Xiaojuan Li, Xingyao Pan, Min Shi, Hang Zhang

**Affiliations:** 1 Key Laboratory of the Ministry of Education Land Subsidence Mechanism and Prevention, Capital Normal University, Beijing, 100048 China; 2 College of Resource Environment and Tourism, Capital Normal University, Beijing, 100048 China; 3 Beijing Laboratory of Water Resources Security, Capital Normal University, Beijing, 100048 China; 4 Beijing Water Sciences and Technology Institute, Beijing, 100048 China

**Keywords:** Environmental sciences, Hydrology, Natural hazards

## Abstract

Land subsidence induced by groundwater level decline has spatiotemporal variations. Taking the Interferometric Synthetic Aperture Radar (InSAR) results and the groundwater subsidence data acquired by the monitoring stations as the source material, this paper aims to reveal the spatiotemporal heterogeneity of groundwater-land subsidence in Beijing plain by using the Wind Rose Map (WRM) method and the Change Point Analysis (CPA) method. The WRM results show that the amount and variation in subsidence differs in different directions. This method detected the formation of new subsidence centers and the slowdown of land subsidence in 2008. The CPA results show that obvious changes are detected in subsidence development at the Wangsiying (WSY), Tianzhu (TZ) and Wangjing (WJ) stations. However, there is a relatively stable trend of groundwater decline and land subsidence at the Tianzhu (TZ) station. The stages of land subsidence development show a significant response to groundwater. Moreover, changes in land subsidence also show delayed response behind the changes in groundwater level. The time-lag could be affected by the variation in amplitude of the groundwater level.

## Introduction

Land subsidence is a geological hazard that could be related to human activities or environmental changes^1^. Land subsidence occurs in more than 150 cities in more than 50 countries in the world, which has become a global environmental geological problem to threaten the human living environment^2–5^. Land subsidence is almost irreversible^6^. Severe land subsidence can decrease the antiseismic performanceor even mangling the infrastructures of buildings^7^, railways^8^, and highways^9^. InSAR technique whichhas the capacity to obtain deformation information over large areas and long periods was widely adopted^10^. The Persistent Scatterer Interferometric Synthetic Aperture Radar (PS-InSAR) technique can achieve a monitoring precision of millimeter level^11^. Other similar approaches, such as the Small Baseline Subset Interferometric Synthetic Aperture Radar (SBAS-InSAR)^12^ and the Interferometric Persistent Target Analysis (IPTA) method^13^, have also been proposed and confirmed to acquire equivalent monitoring results. The PS point dataset, which has a much higher density, can provide a basis for the analysis of the spatial differentiation of land subsidence.

Land subsidence has complex origins, complicated evolutionary processes and different spatiotemporal distributions. The study of the evolution law and the mechanism of land subsidence will help to formulate the corresponding countermeasures, and guide the urban planning and construction, and ensure the regional urban geological safety. The valid description of the spatiotemporal heterogeneity and evolutionary pattern is critical for the understanding of the mechanism of land subsidence. With the InSAR results and other data, many researchers have studied the spatiotemporal pattern of land subsidence. The subsidence in different areas may have different characteristics. There is rapid subsidence in urban areas in western Indonesia which is more serious than other areas^3,14^. The uneven spatiotemporal deformation has caused ground fissures in Xi’an, China^15^.

For a long time, due to the continuous drought and the lack of surface water resources, two-thirds of thewater supply in Beijing is groundwater. As a result, Beijing has experienced long-term overpumping of groundwater^16^. Land subsidence in Beijing is mainly caused by excessive exploitation of groundwater^17,18^. As a result, Beijing becomesone of the most serious cities that suffer from land subsidence in China. The Dongbalizhuang-Dajiaoting subsidence center, the earliest-formedsubsidence centers in Beijing, experienced a subsidence rate of 124 mm/yr during the period from 2003 to 2010^19^. It is reported that wall cracks and pipeline rupturesinduced by uneven land subsidence have been found in Beijing^20^. Land subsidence affects the urban planning, the constructionand the sustainable development of Beijing. The presence of compressible layers is the basis for the development of land subsidence related to groundwater extraction. The differential distribution of groundwater levels in different confined aquifers may lead to the heterogeneous distribution of land subsidence^21^. Areas with large volumes of static and dynamic loads have a significant impact on the distribution of subsidence in uneven ground^22^. Compare withthe variation of groundwater level, most of the changes in land subsidence development have delays in the Beijing area^23^. The lag time between land subsidence and the groundwater level change is 0.57–1.76 months^24^. However, research on the heterogeneity of land subsidence and groundwater is still insufficient. The study of the heterogeneity of land subsidence may help us to better understand the developmental characteristics and enable improved decision making and faster reactions to subsidence.

To better describe and understand the spatiotemporal heterogeneity of land subsidence in Beijing, the authors conducted research with the WRM and CPA methods^25^. A WRM has successfully been used to study the spatial characteristics of public health^26^, the development of atmospheric pollution^27,28^ and the spatiotemporal variation in climate change^29,30^. For time-series data, many scientists have carried out the weather change detection research^31^, damage detection research^32^ and medical research^33^ by using the CPA technique. There are periodic and abnormal signal in time-series groundwater level data. However, in time-series land subsidence data, there is less periodic and abnormal signals, but stronger trend signals. The periodic and abnormal signals would affected the determination of the change points. As a result, different change points may obtained for different types of data, which can affect the analysis of the relationship of the two dada set in this article. We intend to find the stage changes in the time-series subsidence and groundwater data. Different stages between the changes may have different development trend, which can reflect the development process of land subsidence and groundwater level variations. These changes should not occur at the peaks and valleys of a time-series data, but between two statistically different stages. On that basis, we can analyze the relationship of the change points between the two different types of data. CPA method can detect the statistical change points. What is more, the confidence level and the confidence interval of the changes are estimated by CPA method, which can better describe the detected changes and further improve the reliability of the detection results^25^. By analyzing the change points of land subsidence data and groundwater level data, we come to some new conclusions.

The objectives of this study are firstly, to analyzes the spatiotemporal heterogeneity of ground subsidence with the WRM and CPA method. Secondly, to examine the directional characteristics in different time periods. Thirdly, to validate the relationship of the develop stages of land subsidence and groundwater. The paper is organized as follows. “Methods and materials” section describes the study area, the datasets and methods. “Data analysis methods” section verifies and analyzes the results. Detailed analysis and discussion of the results are given in “Results” section.

## Methods and materials

### Study area

**Figure 1:**
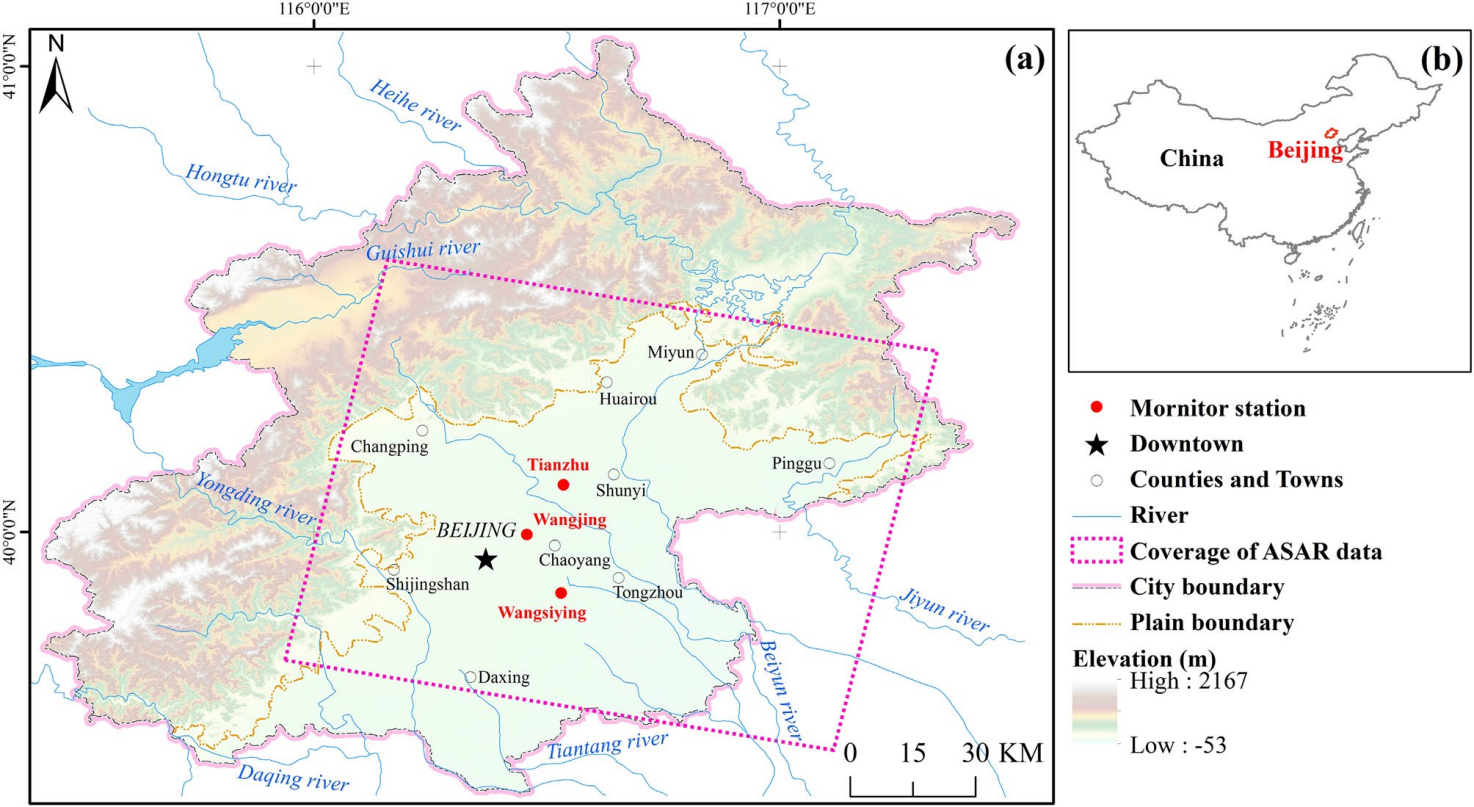
Location of the study area. (**a**) The pink dashed line shows the spatial coverage of Envisat ASAR data, which can cover most of the Beijing Plain area. The red dots represent the subsidence monitoring stations. (**b**) Location of Beijing. (Figure generated using ArcGIS v.10.6.0.8321, www.esri.com).

Beijing, the capital of China, covers an area of approximately 1.64 × 10^4^ km^2^ (Fig. 1). The elevation is high in the northwest and low in the southeast. The elevation is between 20 and 60 m in the plain area. There are continuous mountains in western and northern Beijing and a vast plain in southeastern Beijing. The plain area accounts for approximately 38% of the total area^34^. The Beijing Plain was formed by the alluvial-diluvial processes of the Yongding River, Chaobai River, Ju River, Wenyu River and Juma River. These alluvial fans are staggered and intersected, which makes the lithology of the vadose zone and the aquifer complicated. From the front of the mountain to the plain, the thickness of the deposition gradually increases, and the aquifer material changes from coarse to fine. At the same time, the structure of the aquifer changes from a single gravel stratum to a multilayer aquifer system with sand and clay.

Beijing has a warm temperate semihumid and semiarid continental monsoon climate. The average rainfall is 537.2 mm (1978–2010)^35^. Beijing has suffered a continuous drought since 1999. The urbanization accelerated significantly during this period, and the population rose from 12.572 million to 19.612 million^35^. Groundwater was seriously overexploited as the main water source. It is reported that two-thirds of the water demand was supplied through groundwater wells from 2004 to 2013^34^. According to statistics, the average annual overexploitation of groundwater from 2001 to 2010 was 685 million m^3^/yr^35^. As a result, land subsidence rapidly developed in the plain areas such as Shahe-Baxianzhuang area, Chaoyang district, Sourthern Shunyi, Western Tongzhou and Sourthern Daxing in this period^36^. The subsidence area with a rate greater than 50 mm/yr was 265.41 km^2^ until 2010^37^.

### Materials

#### SAR data

In this paper, 36 Envisat-ASAR (Advanced Synthetic Aperture Radar) images acquired from June 2003 to November 2010 are used to extract the deformation information (the coverage of Envisat-ASAR dataset is shown in Fig. 1). The Envisat satellite is one of Earth observation satellites launched by the European Space Agency on March 1, 2002. It is a sun-synchronous orbit satellite. ASAR, the largest equipment carried by Envisat, works in C-band with a wavelength of 5.6 cm. The incidence angle is approximately 22.8° to 22.9°. The parameters of the ASAR images utilized are listed in Table 1.

**Table 1:** Parameters of the SAR data used.

Parameter	Envisat ASAR
Orbital altitude	799.8 km
Band	C
Wavelength	5.6 cm
Polarization	VV
Orbit direction	Ascending
Repeat observation period	35 days
Spatial resolution	30 m
Number of images	36
Date range	June 2003–November 2010

#### Layered deformation and groundwater monitoring data

This study collected three sets of deformation and groundwater monitoring data obtained by three land subsidence monitoring stations in Beijing (the location can be seen in Fig. 1). The subsidence monitoring stations include the WSY station, WJ station and TZ station. There are several borehole extensometers in each station to monitor the deformation at different depths. A series of boreholes with different observation depths are set to monitor the groundwater levels. In this paper, the time-series data from extensometers and the water-level observation wells were acquired from January 2004 to November 2010, and the sampling interval was 15 days.

**Figure 2:**
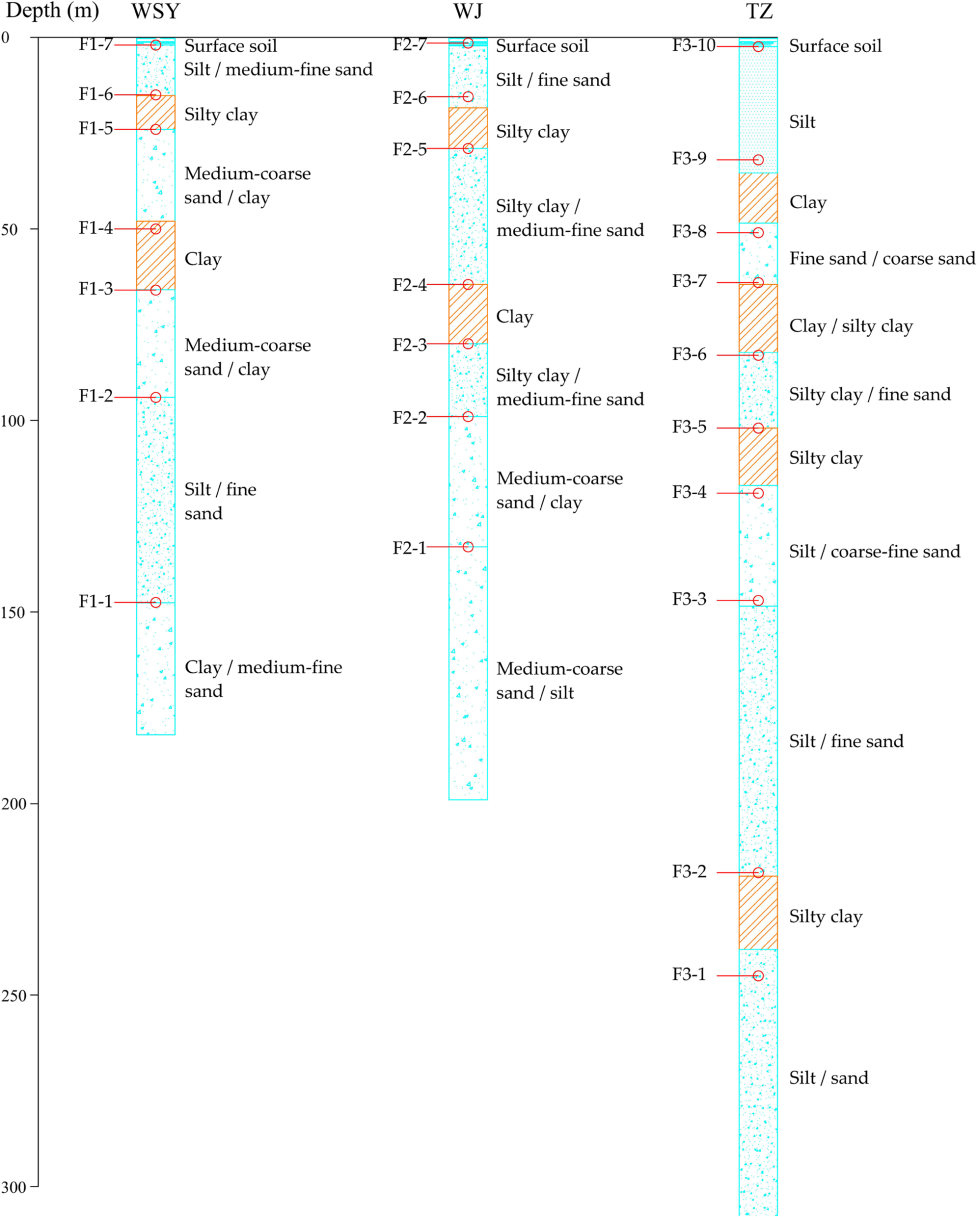
The observation layers of the extensometers and the corresponding lithology of the three monitoring stations.

There are ten extensometers at the TZ station and seven extensometers at the WSY and WJ stations. For groundwater level monitoring, six observation holes are set at the TZ station, and five observation holes are set at the WSY and WJ stations. It has been reported that the first and second confined aquifers, with a bottom depth of approximately 100 m, have the largest impact on the spatial evolution of land subsidence in the Beijing Plain^21^. Therefore, groundwater and subsidence monitoring data with a depth of approximately 100 m were selected to study the temporal evolution of groundwater and land subsidence. The extensometer F1-3 (WSY) can monitor subsidence at depths from 65.9 to 94 m. The monitoring depth is from 80 to 99 m for F2-3 (WJ) and from 64. 5 to 82.3 m for F3-7 (TZ). The observation depths are 64.9 m ~ 94 m, 79.86 ~ 96.88 m and 85.7 ~ 91.3 m for D1-3 (WSY), D2-3 (WJ) and D3-4 (TZ), respectively. The observation layers of the extensometers and the corresponding lithology of the three monitoring stations are described in Fig. 2, and detailed information is listed in “Supplementary file”.

## Data analysis methods

### Interferometric point target analysis (IPTA)

The signal of each pixel in a SAR dataset contains two components: the real and imaginary parts. The real component reflects the reflection intensity of the ground surface to the radar beam. The imaginary component is the phase value related to the radar slant range. The phase value is the main information used in the InSAR process to estimate ground deformation. For example, the main PS-InSAR process consists of two stages, namely, PS identification and phase analysis. The differential phase of each PS point can be extracted by the Differential Interferometric Synthetic Aperture Radar (D-InSAR) method, and surface deformation can then be determined after the removal of disturbances from the differential phases.

First, a master image is selected and registered with other slave images. Then, we can obtain all interferograms after a complex conjugate multiplication between the master image and each slave image. The differential interferograms are achieved by subtracting the topography phase, which is simulated from the digital elevation model (DEM).

By combining amplitude dispersion and the correlation between amplitude variance and phase stability, IPTA can identify some stable pixels even in nonurban areas. The phase of every PS target in one interferogram can be abstracted as four components. For the x-th PS point in the i-th differential interferogram, the phase can be represented as Eq. (1):

1 $$\varphi_{int,x,i} = {\varphi}_{def,x,i} + {\varphi}_{\varepsilon ,x,i} + {\varphi}_{atm,x,i} + {\varphi}_{n,x,i}$$

where *φ*_*def*_ is the deformation phase at the Line Of Sight (LOS) of radar; *φ*_*ε*_ is the residual terrain phase; *φ*_*atm*_ is the atmospheric phase delay; and *φ*_*n*_ is random noise.

The surface deformation is assessed by dividing it into linear and nonlinear deformation, and then the differential phase can be represented as Eq. (2):

2 $$\varphi_{int,x,i} = {\varphi}_{linear,x,i} + {\varphi}_{\varepsilon ,x,i} + {\varphi}_{res,x,i}$$

where *φ*_*linear*_ is the linear deformation phase; *φ*_*res*_ is the residual phase, including the atmospheric affect *φ*_*atm*_, nonlinear deformation *φ*_*nonlinear*_ and noise *φ*_*n*_.

To obtain the linear deformation component, a phase difference calculation is carried out between every neighborhood PS point, which results in the variation of the topographic phase error ${\Delta}$*φ*_*ε*_, the variation of linear deformation phase ${\Delta}$*φ*_*linear*_ and the variation of residual phase between them ${\Delta}$*φ*_*res*_. Generally, it is most likely that the |${\Delta}$*φ*_*res*_|< π, and ${\Delta}$*φ*_*linear*_ and ${\Delta}$*φ*_*ε*_ can be estimated from the wrapped phase of each interferogram^38^. With the established PS network, the adjustment method can be used to estimate the linear deformation *φ*_*linear*_ and residual terrain phase *φ*_*ε*_ of each PS point. According to formula (2), we can calculate the residual phase for each PS point. To obtain the complete deformation information of each PS target, a phase analysis is needed to separate the nonlinear deformation component from the residual phase.

The atmospheric component has little temporal correlation but strong spatial correlation. From the perspective of signal analysis, the phase due to atmospheric delay shows high-frequency characteristics on a time series; however, it has low-frequency characteristics in the spatial domain. The ground deformation, which has both spatial and temporal correlations, shows low-frequency characteristics in the space domain and time domain. The noise has neither spatial nor temporal correlations.

The characteristics of the components in the frequency domain enable us to distinguish the components. First, temporal high-pass filtering of the residual phase of each PS point is conducted, resulting in a combination of atmospheric and noise phase components. Next, spatial low-pass filtering is performed on each of the interferograms to remove the noise phase and obtain the atmospheric phase of all PS targets on each differential interferogram. Due to the low correlation between atmospheric conditions and time, the atmospheric phase of the master image can be the average of the residual phase of each PS point in the time series^11^. Then, with the atmospheric phase of all differential interferograms and the master image, the atmospheric phase of all slave images can be obtained by determining the difference between the above two.

Finally, the atmospheric phase of each image is subtracted from the original residual phase φ_res_, and temporal low-pass filtering is applied to obtain the nonlinear deformation phase of all PS points corresponding to each interferogram. By adding the previously acquired nonlinear phase to the linear phase, we can obtain the ultimate time-series deformation of all PS targets. The InSAR technique utilized measurements from signals between sensors and ground features. The LOS deformation produced by the PS-InSAR technique has been suggested to achieve mm accuracy^39,40^. Then, the vertical deformation can be extracted from the LOS deformation according to the incidence angle of the sensor.

### Wind rose map (WRM)

A WRM is a professional statistical chart used to quantitatively analyze wind elements and is used for the analysis of wind speed and power characteristics. It has also been used to study the spatial evolutionary pattern of urban development and urban green land. It can express the spatial heterogeneity of a certain characteristic in different directions and the change of a certain characteristic at different times. The method can be implemented by taking a certain point as the origin and dividing the two-dimensional space into several quadrants (there are sixteen quadrants in this paper with a dividing angle of 22.5°). Then, the statistical information in each quadrant can be studied.

A WRM is used in this paper to quantitatively study the subsidence in Beijing from 2003 to 2010. The article takes the Palace Museum, which is known as the center of Beijing, as the origin to carry out the WRM analysis. The study sets a radius of 80 km, which can cover all the areas of the Beijing Plain. Then, the study area is divided into sixteen equal subregions. The subsidence characteristics represented by PS points in each subregion are statistically studied by means of the mean value and the standard deviation (Eq. 3) of subsidence in each year.

3 $$PS_{sd} = \sqrt {\frac{1}{N}\mathop \sum \limits_{i}^{N} \left( {PS_{i} - \overline{PS} } \right)^{2} }$$

where *PS*_*i*_ is a point in a subregion, $\overline{PS}$ is the mean value of all points in the subregion,* N* is the number of PS points in the subregion, and *PS*_*sd*_ is the standard deviation of all PS points in the subregion.

The two methods can be used to analyze the condition related to land subsidence development in the different periods. The results are expressed as wind rose maps to intuitively present the differences in the intensity of ground settlement expansion in different subregions during different periods. Moreover, these maps aim to distinguish the dominant direction of urban land subsidence extension at different times. Each subregion has an equal area but a different direction, and the resulting spatial and temporal characteristics of land subsidence have strong comparability.

### Change point analysis (CPA)

The discovery of change points is of great significance in time series analysis. There are many detection methods for change points. Among them, the cumulative and control graph-based method called Cumulative Summation (CUSUM) has simple principles and high efficiency. The cumulative sum is the sum of all the differences between all original values and their mean. A sudden change in the direction of the CUSUM map indicates that the time series has changed. The method is based on the sequential probability ratio test, which gives the method a high sensitivity and strong anti-risk capacity.

The CPA technique is implemented by repeating the CUSUM and bootstrapping methods^25^. The purpose of the CPA method used in this paper is mainly to determine whether a change has occurred within the time series, and it can detect the small changes omitted by the control graph. The bootstrap analysis is introduced into the evaluation to increase the confidence level. By comparing the confidence level and the confidence interval, this method can better describe the detected changes and further improve the reliability of the detection results of the change points.

The CPA technique is implemented by repeating the CUSUM and bootstrapping method. The CUSUM should be calculated first. Assuming N time series data *X*_*1*_*, X*_*2*_*, …, X*_*N*_*, N* cumulative sum *S*_*0*_*, S*_*1*_*, …, S*_*N*_*,* can be calculated. The specific steps are: 1. The average $\overline{X}$ = (*X*_*1*_ + *X*_*2*_ + … + *X*_*N*_)/*N*; 2. The initial cumulative sum is set as 0, *S*_*0*_ = 0; 3. The subsequent cumulative sum is the previous cumulative sum plus the difference between the current value and the mean; that is, for *i* = 1,2, ……, *N*, the cumulative sum is *S*_*i*_ = *S*_*i*_ − 1 + (*X*_*i*_ − $\overline{X}$). The cumulative sum is not the sum of all the original values but the sum of all the differences between all original values and the mean. Since the differences in the sum of all the original values and the sum of the corresponding mean should be zero, *S*_*N*_ = 0.

Assuming that the time series data values are larger than the mean over a certain period, the CUSUM graph exhibits a steady increasing trend with a positive slope. Likewise, if a slope is negative, it shows that the original value is less than the mean. A sudden change in the direction of the CUSUM map indicates that the time series has changed. After a change point is detected, it is set to the first level. Then, the time series can be segmented according to the position of the catastrophe point, and the catastrophe point of the subsequence can be detected as before. Theoretically, there is at most one catastrophe point at the first level, two at the second level, four at the third level, and 2(*N*−1) at the *N*th level.

Although the CUSUM diagram can show a change, it does not indicate whether the change has occurred or show the confidence level. Thus, the bootstrap analysis is introduced into the evaluation of the confidence level. First, the difference between the maximum CUSUM and the minimum CUSUM of the original sequence is used as the range of variation, which is

4 $$S_{diff} = \, S_{max} - \, S_{min}$$

Then, the original time series data X_1_, X_2_, …, X_N_, are sorted randomly to generate N bootstrap samples *X*_*1*_^*0*^*, X*_*2*_^*0*^*, …, X*_*N*_^*0*^. Recalculate the CUSUM diagram of the bootstrap samples and the corresponding variation range *S*_*diff*_^*0*^. Then, *S*_*diff*_^*0*^and *S*_*diff*_ are compared to complete a bootstrap. Bootstrap verification is a process of comparing many bootstrap outcomes with the original time series. Let *A* be the number of bootstraps performed and let *B* be the number of bootstraps for which *S*_*diff*_^*0*^ and *S*_*diff*_, and the confidence level can be calculated as Eq. (5):

5 $$Confidence\, level = \frac{B}{A}*100\%$$

There is another indicator named the level used by the CPA method to describe the importance of the change. Level 1 changes are detected on the first pass through the data, and level 2 changes are detected on a second pass through the data. To obtain better results by using the above method, the time-series data should have the same interval within each sequencing dataset. According to the above theory, the groundwater level and land subsidence change process of three land subsidence monitoring stations are analyzed.

## Results

### Time series deformation result by IPTA

**Figure 3:**
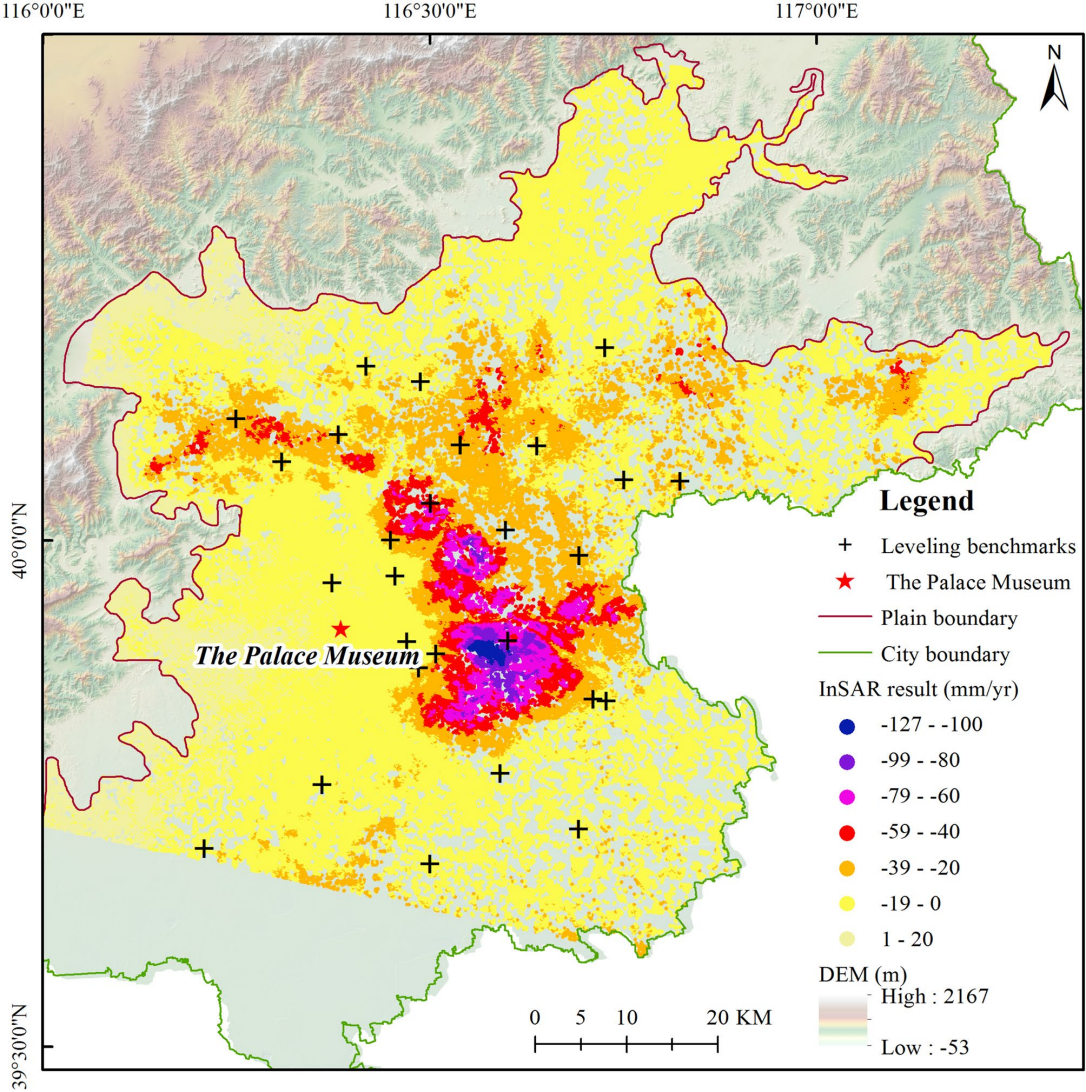
Land subsidence rate from June 2003 to November 2010 extracted by the IPTA method. The red star is the location of the Palace Museum, which is known as the center of Beijing. The article takes the Palace Museum as the center to carry out the WRM analysis. The black crosses are the locations of leveling benchmarks for the validation of the InSAR result. (Figure generated using ArcGIS v.10.6.0.8321, www.esri.com).

The IPTA method is used to extract the subsidence information of the study area during 2003 and 2010 (Fig. 3). There were 267 thousand PS points calculated in the results. The PS density obtained by ASAR is approximately 27 points per square kilometer. From June 2003 to September 2010, the maximum cumulative subsidence amount and the maximum subsiding rate of 900 mm and 127 mm/yr, respectively, occurred in the Dongbalizhuang-Dajiaoting land subsidence center. The spatial distribution characteristics of the subsidence rate in Beijing from 2003 to 2010 are consistent and appear uneven. The main subsidence areas and the subsidence rates agree with previous research results^41^.

**Figure 4:**
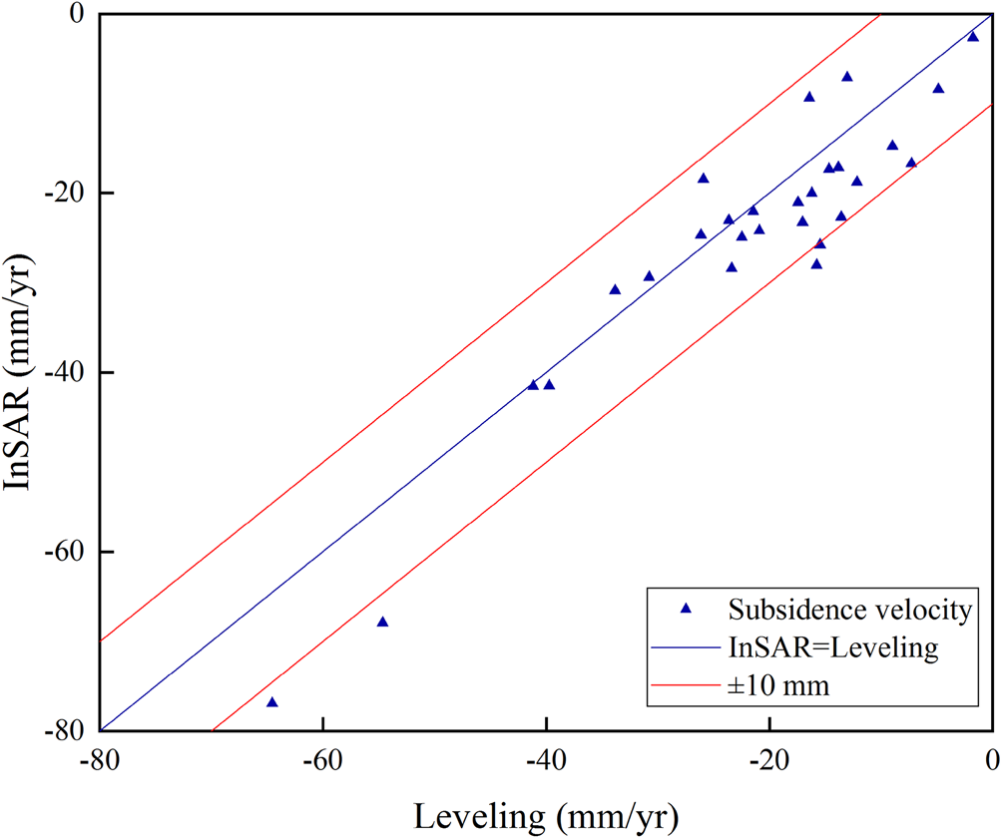
Validation of IPTA result.

A leveling survey is the most effective method to measure the ground deformation with high precision. The second-order leveling results of twenty-eight leveling benchmarks are collected to verify the InSAR results in this paper. The mean value of the nearest ten PS points around each leveling benchmark is compared with the leveling data. The results show that the maximum error is − 13.3 mm, the minimum error is 0.3 mm, the mean error is − 3.2 mm, and the Root Mean Square Error (RMSE) of the error is 5.6 mm (Fig. 4).

### Spatial variation pattern of subsidence

**Figure 5:**
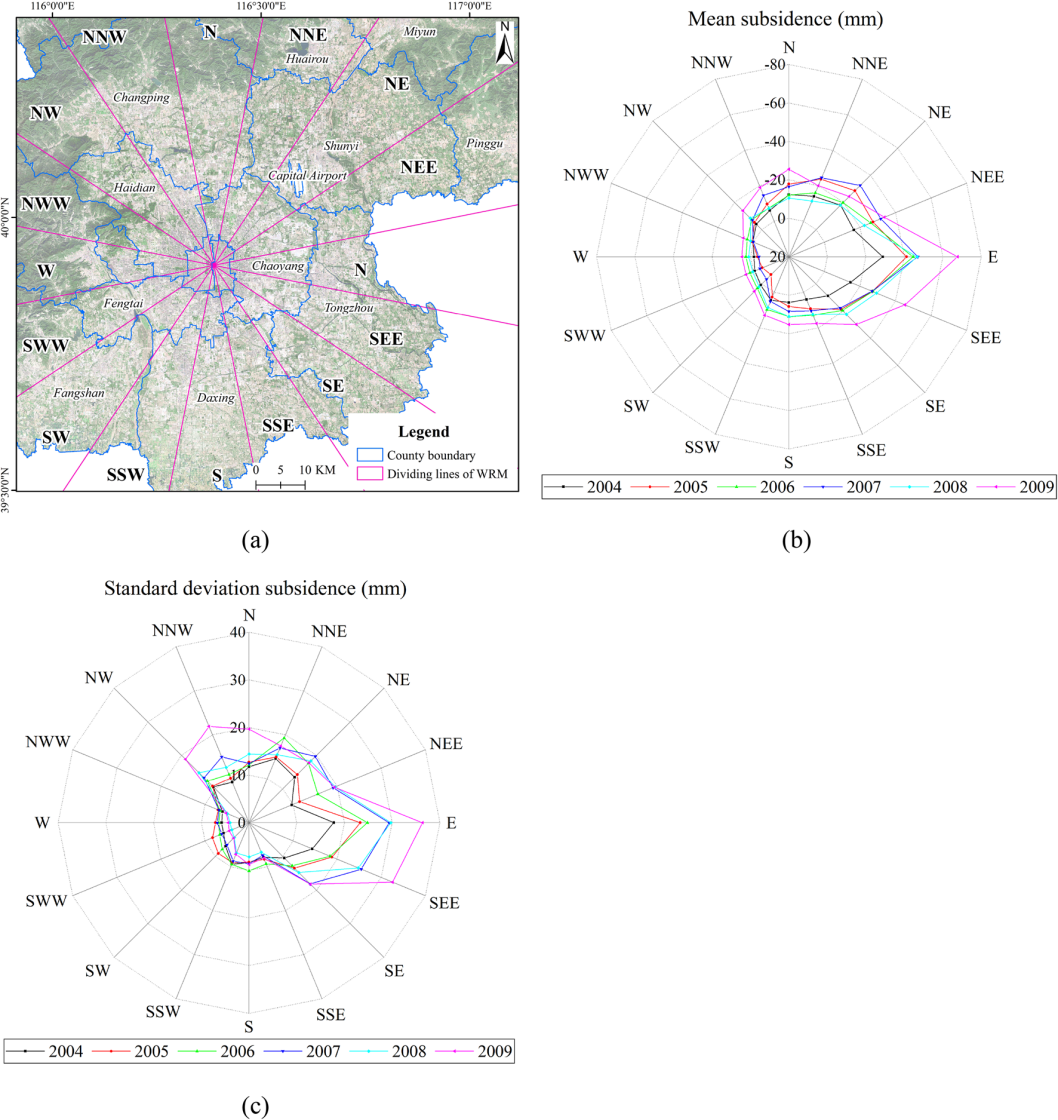
(**a**) WRM map of the mean subsidence in each subregion from 2004 to 2009. (Figure generated using ArcGIS v.10.6.0.8321, www.esri.com) (**b**) WRM map of the mean subsidence in each subregion from 2004 to 2009. (**c**) WRM map of the standard deviation subsidence in each subregion from 2004 to 2009.

The spatial variation pattern of subsidence can be analyzed from the WRM. The Beijing area is divided into sixteen quadrants (Fig. 5a). The land subsidence in each direction is expressed by the mean subsidence quantity of all the PS points in the corresponding region from 2004 to 2009. There were significant differences in land subsidence in different directions at the same time, and there were also obvious differences in the development of subsidence in different time periods in the same direction. The WRM is used to better explain the differences in land subsidence during each period and to better analyze the trend of land subsidence development and the diffusion and agglomeration characteristics of ground subsidence (Fig. 5b). The difference in land subsidence in each direction was based on the average of all PS points located within the corresponding subregion. There is an obvious difference in land subsidence in different time periods, which shows a certain directional characteristic. From 2004 to 2009, the development of the annual settlement in each direction was different. The amount of land subsidence significantly increased in some directions, such as E, SEE and SE, which further highlights the difference in spatial subsidence development in each direction. Particularly, the subsidence has a trend of agglomeration in the E and SEE directions. The E and SEE subregions cover the Dongbalizhuang-Dajiaoting subsidence center, which was the center of industrial development the early days. The nature of subsidence also presents a staged development; it grew rapidly before 2008, stagnated or even decreased in 2008, and then progressed after 2008.

The standard deviation of subsidence in each subregion can be described in Fig. 5c. The difference in PS points within each subregion varies greatly. The subsidence in the NW, NNW, E and SEE subregions has larger differences, which also grew rapidly. The growth of the standard deviation indicates the formation and deterioration of the subsidence centers. Figure 5b and c show that the subsidence quantity and acceleration are large in the E and SEE subregions. While the amount of subsidence was relatively small, subsidence acceleration was high in the N, NNW, and NW. The E and SEE subregions cover the Central Business District (CBD) area, which is currently an economic development center. The population density is high, and the surface load brought by super high-rise buildings is more concentrated than in other regions.

### Stage development of land subsidence

The above research reflects the overall development of land subsidence during 2003–2009. Due to the poor time interval of Envisat ASAR acquisitions, it is not clear whether there are multistage developmental features of land subsidence. Therefore, this paper selects the ground subsidence data of three land subsidence observation stations for further analysis. CPA is mainly used to analyze the stage characteristics. With the change points detected by the CPA method, we try to understand the temporal evolutionary pattern of land subsidence.

**Figure 6:**
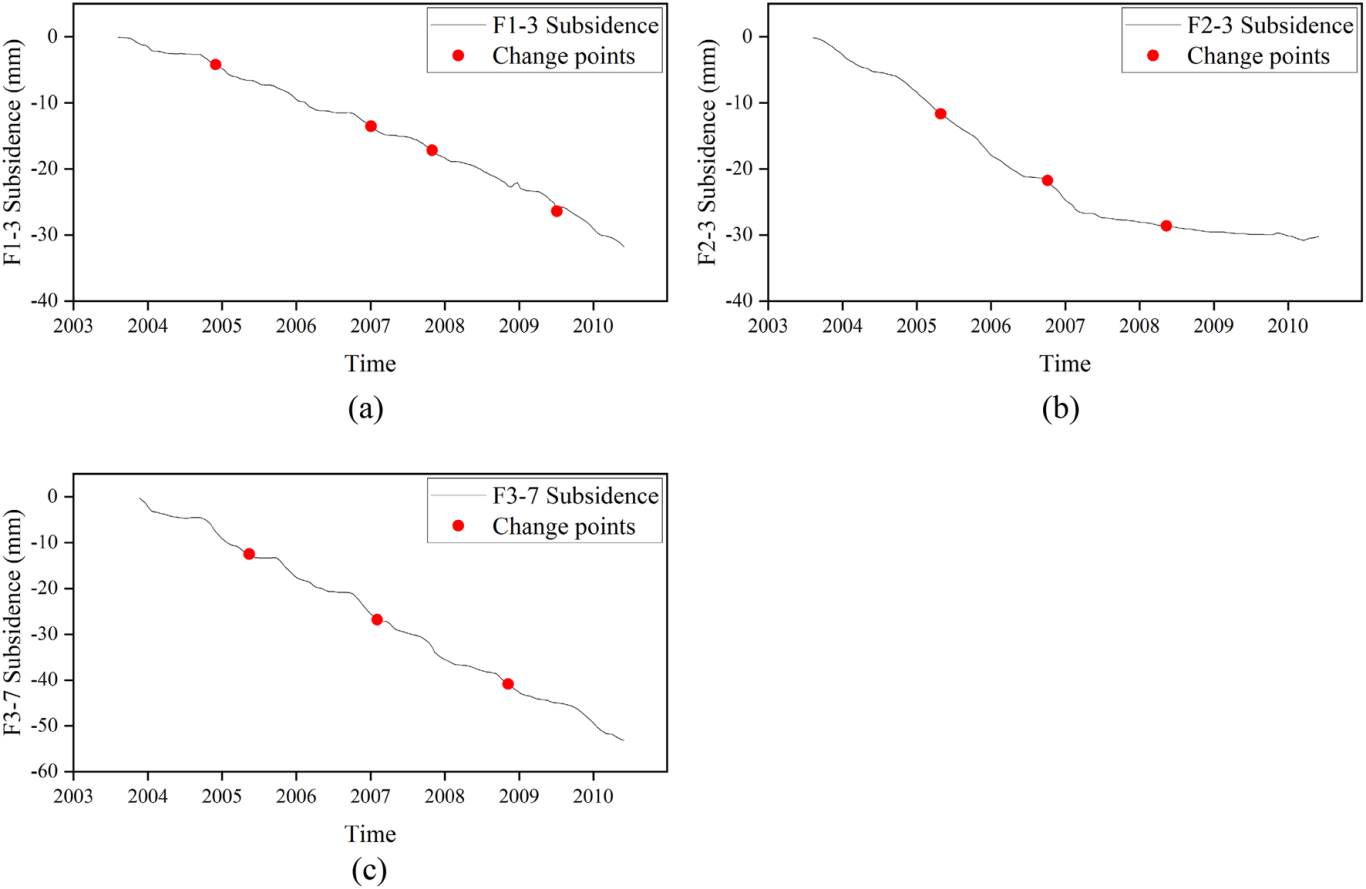
The detected change points result for subsidence. The red dots represent the change points of subsidence. (**a**) Change points of F1-3 at WSY station. (**b**) Change points of F2-3 at WJ station. (**c**) Change points of F3-7 at the TZ station.

The change points of the land subsidence are demonstrated in Fig. 6. From January 2004 to November 2010, four change points are detected in F1-3 at the WSY station (Fig. 6a). Three change points are detected in F2-3 at the WJ station during the same period (Fig. 6b). Three change points were detected in F3-7 at the TZ station (Fig. 6c). The subsidence of the three sites showed a relatively steady rate from 2004 to 2007. The subsidence velocity decrease at F2-3, and the other two sites continued to subside stably. All the change points at the three stations have confidence levels greater than 90%.

## Discussion

### The directional characteristics in different time periods

**Figure 7:**
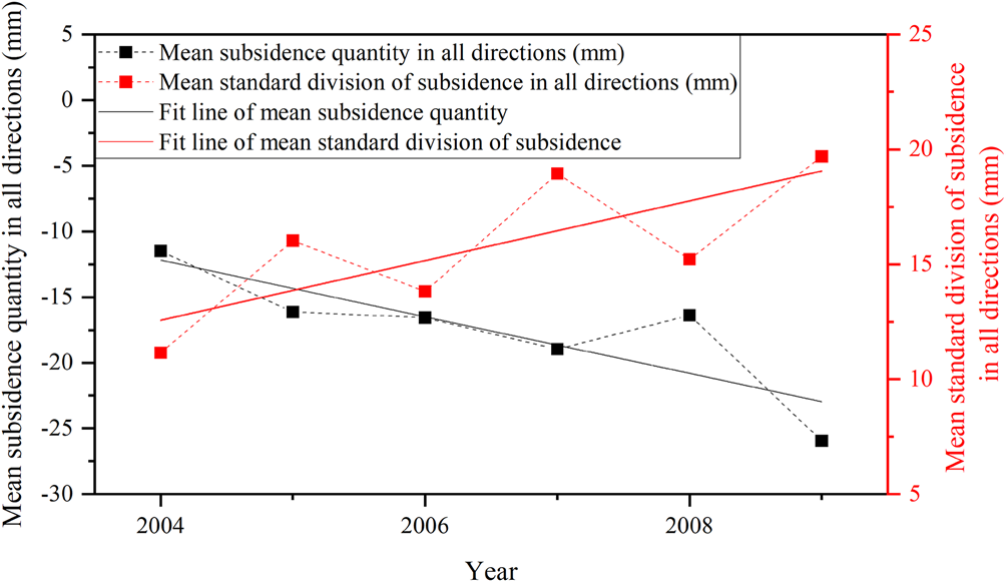
The time-series mean amount of subsidence and mean standard division of subsidence in all directions. The dashed black line is the time-series mean amount of subsidence in all directions. The dashed red lineis the time-series mean standard division of subsidence in all directions. The solid black line is the linear fit of the mean subsidence. The solid red line is the linear fit of the mean standard division of subsidence.

The WRM method can identify the developmental characteristics of land subsidence in different directions (Fig. 5b and c). The result of this method is related to the location of the center. In this study, there is a great difference in land subsidence in different azimuths. It developed rapidly in the E, SEE, and SE during the study period, which can reflect the rapid development of the subsidence funnel in the Chaoyang and Tongzhou districts. The standard deviation of land subsidence has shown a rapid growth trend along the NW–SE direction, which can reflect the development of uneven subsidence Changping funnel and Chaoyang funnel. The groundwater in these areas has been overexploited for a long time, and the groundwater level has dropped rapidly. Land subsidence in such areas needs more attention. With the WRM method, we can also obtain the differences in other characteristics in all directions in different time periods, including the mean amount of subsidence and standard deviation. Such indicators are mainly analyzed by combining Fig. 5b and c. Figure 7 shows that the amount of subsidence accelerated obviously from − 11.48 mm in 2004 to − 25.95 mm in 2009. At the same time, the standard division also showed an increasing trend. However, in 2008, the mean amount of land subsidence in all directions was − 16.38 mm, which was lower than that in the previous year and the following year.

**Figure 8:**
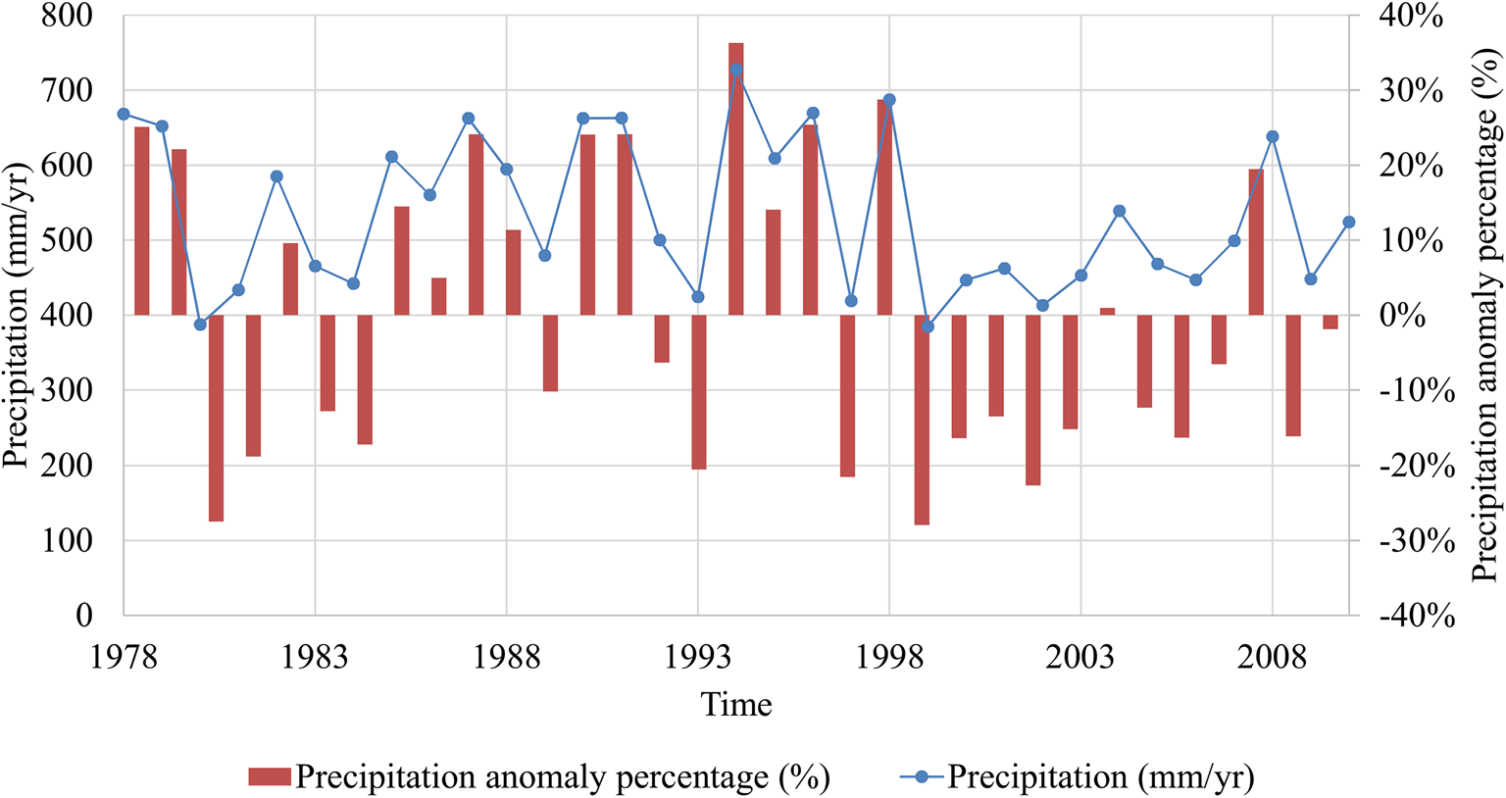
Average annual precipitation in the Beijing area from 1978 to 2010.

**Figure 9:**
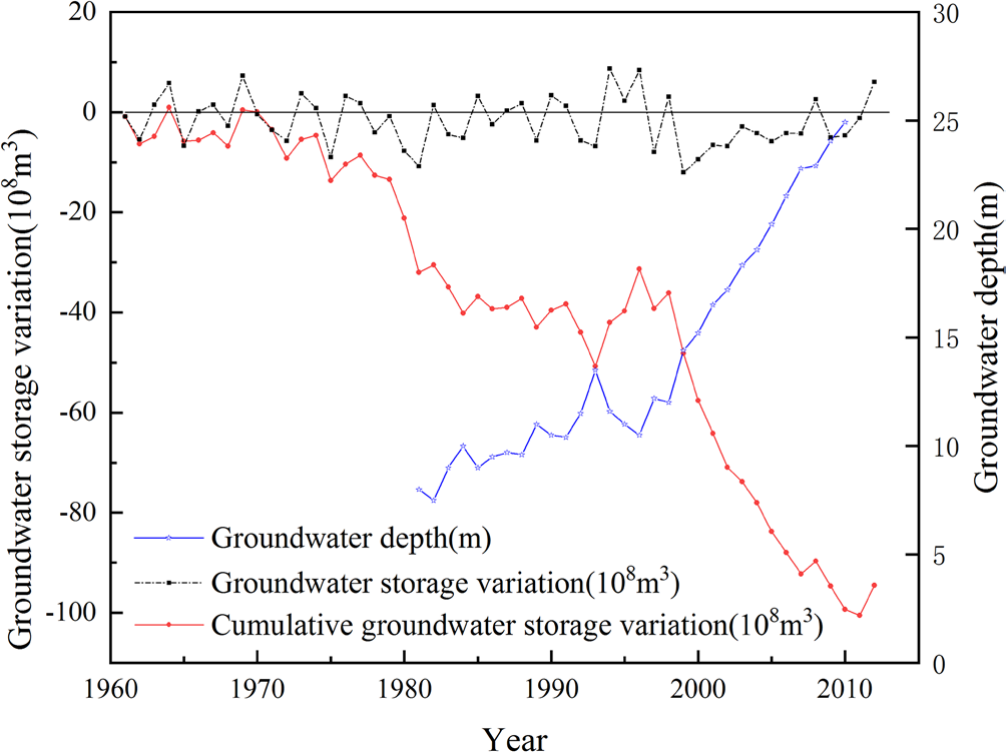
Groundwater storage and depth variation in Beijing area. The horizontal black solid line indicates that the groundwater storage variation is zero.

To analyze what happened in 2008, precipitation data (Fig. 8), groundwater depth data and groundwater storage data (Fig. 9) were collected from reference^35^. The precipitation anomaly percentage is used to describe the division of the precipitation in a certain year from the mean annual precipitation of a period. It can be represented as the following equations:

6 $$P_{a} \left( \% \right) = \frac{{P - \overline{P}}}{P} \times 100\%$$

7 $$\overline{P} = \frac{1}{n}\mathop \sum \limits_{i = 1}^{n} P_{i}$$

where *P*_*a*_*(%)* is the precipitation anomaly percentage; *P* is the precipitation of a certain year; $\overline{P}$ is the mean annual precipitation of a period; and *n* is the number of years in the study period, *i* = 1, 2, 3…*n*.

Figure 8 shows that from 1999 to 2010, Beijing suffered continuous drought. From the precipitation curve, the rainfall in 2008 was above 600 mm, and rainfall in most of the recent years was below 500 mm. The histogram of the precipitation anomaly percentage shows that the precipitation in 2008 was 100 mm higher than the average. The precipitation in 2004 and 2010 was close to the average, and in other years, it was nearly 100 mm below the average since 1999. This plays a very important role in the replenishment of groundwater and indirectly affects the development of land subsidence. To clarify the impact of rainfall, the groundwater storage variation and the groundwater depth data are also described in Fig. 9.

The continuous drought has caused long-term insufficient groundwater recharge. During this period, the groundwater storage variation was negative, except in 2008 and 2012, which made the cumulative groundwater storage variation decrease rapidly. This reduction in groundwater storage is reflected by the increase in groundwater depth. The subsidence in different directions shows different responses to groundwater variation. Subsidence in most directions presented a decreasing tendency in 2008.

The WRM method detects not only the directional differences of land subsidence, but also the variation in the subsidence development. Spatially, the subsidence develops differently in each direction. Temporally, the subsidence would be affected by the precipitation variation and groundwater level change.

### Temporal evolution pattern of land subsidence and groundwater by the CPA method

**Figure 10:**
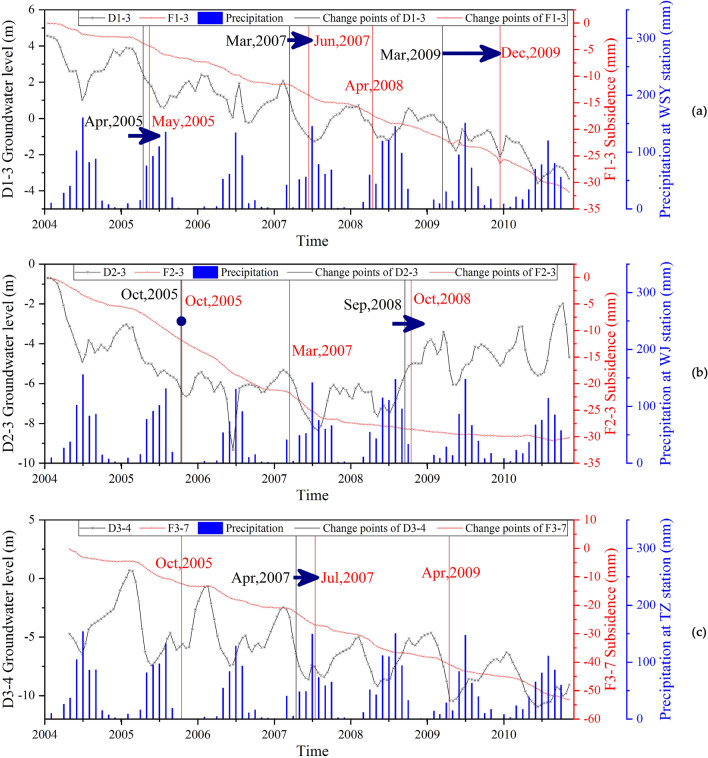
The relationship between the change points of the groundwater level and subsidence. The vertical black solid lines indicate the location of the change points of the groundwater level. The vertical red solid lines show the location of the change points of land subsidence. (**a**) Change points of D1-3 and F1-3 at WSY station. (**b**) Change points of D2-3 and F2-3 at WJ station. (**c**) Change points of D3-4 and F3-7 at TZ station.

The results of the CPA show staged changes in the characteristics of groundwater level and land subsidence development. The time-series data of different sites also show some differences. There are obvious staged characteristics of the changes in groundwater level and subsidence at the WSY (Fig. 10a) and WJ (Fig. 10b) monitoring stations. The groundwater level at the TZ (Fig. 10c) monitoring station showed a fluctuating decreasing trend. Similarly, the deformation velocity remained stable during the period.

Based on the above quantitative and qualitative analysis, the variations in groundwater level and subsidence both have staged features. The number and occurrence time of changes are different between the two kinds of time-series data. However, there is a certain correlation among the change points between the groundwater level and the land subsidence. The changes in subsidence all occurred after changes in groundwater changes. The maximum delay of subsidence is 6 months, and the minimum delay is 1 month.

There are some differences in the changing characteristics at different monitoring stations. For the WSY station, the fluctuation range of the groundwater level continues to decrease during this period. It is more difficult for a smaller groundwater level change to cause a change in the trend of land subsidence. In this case, the delay of land subsidence relative to the change in groundwater level will also increase. Therefore, the delay gradually increased from 1 to 3 months and then to 6 months.

For WJ station, D2-3 is divided into three stages: the decreasing stage, the stable stage and the recovery stage. F2-3 is mainly divided into four development stages. The first two stages have a rapid development of land subsidence, and the slope of the subsidence curve is relatively large. From the third stage, the subsidence rate begins to decrease sharply, and by the fourth stage, the subsidence rate is relatively low. The first and third change points of F2-3 at WJ Station are consistent with the two change points of D2-3. The second change point of F2-3 occurs during a period when the groundwater level is relatively stable. The first land subsidence change occurred during almost the same period as the groundwater level. The second land subsidence change occurs 1 month after the groundwater change. The response of the ground subsidence trend to the groundwater level trend is obvious. In the first stage, when the groundwater level dropped sharply, the land subsidence rate was relatively high. In the first half of the stable stage of the groundwater level, the land subsidence rate was still large, and the subsidence rate began to slow in the second half of the stable stage. When the groundwater level was rising, the land was still subsiding but at a very low velocity.

The groundwater level and subsidence at the TZ station have similar trends as those at the WSY station. F3-7 has basically the same trend in all four stages. The second change point of F3-7 is consistent with the only change point of D3-4, with a delay of 3 months. There are no other corresponding change points detected in the groundwater level data. Land subsidence shows a stable development state when the groundwater level has a stable trend.

Statistically, the changes detected in the time series groundwater level and land subsidence mainly occurred in 2005, 2007, 2008 and 2009. The detected changes in the water level are less than the subsidence due to the influence of the fluctuation character of the water level. There is some association among the change points of the groundwater level and land subsidence at the three stations (Tables 2 and 3). Both D1-3 and D2-3 detected a change in 2005, and D1-3 and D3-4 detected a change in 2007. Correspondingly, all three detected time-series subsidence change points in 2005 and 2007. However, there were some differences among the changes in 2008 and 2009. The development of land subsidence had some similar characteristics and some differences among the three stations.

**Table 2:** Change points of groundwater level boreholes.

Groundwater level borehole ID	D1-3	D2-3	D3-4
Observation depth	64.9–94	79.86–96.88	85.7–91.3
Changes	Apr, 2005	Oct, 2005	
	Mar, 2007		Apr, 2007
		Sep, 2008	
	Mar, 2009

**Table 3:** Change points of extensometers.

Extensometer ID	F1-3	F2-3	F3-7
Observation depth	65.9–94	80–99	64.5–82.3
Changes	May, 2005	Oct, 2005	Oct, 2005
	Jun, 2007	Mar, 2007	Jul, 2007
	Apr, 2008	Oct, 2008	
	Dec, 2009		Apr, 2009

The subsidence showed a strong response to the variation in groundwater with a delay of several months. When the groundwater varies greatly, the response of land subsidence within a predicable period of time. The amount of delay has a certain relationship with the amplitude of variation of the groundwater level. The staged characteristics of the subsidence were also affected by the hydrogeological conditions of the three monitoring stations, which have interbed aquifer and impermeable aquifer structures. According to the soil properties, the water release and deformation of the clay layer is slow and will occur behind the groundwater decline. When the water level rises, the resilience of the impermeable layer and the sand layer also show different characteristics. The resilience of the sand layer is basically synchronized with the groundwater level. However, the replenishment of the pore water of the impermeable layer is slow and will take a longer time. Therefore, a noticeable rebound of the impermeable layer does not occur after the groundwater level rises.

## Supplementary Information

Supplementary Information.
