# Supplementary material for: Spatiotemporal heterogeneity of land subsidence in Beijing
Source: Sci Rep. 2022 Sep 6;12:15120. doi: 10.1038/s41598-022-16674-6 (PMC9448723; doi:10.1038/s41598-022-16674-6)
Supplement: Supplementary file 1 — Supplementary Information. [file 41598_2022_16674_MOESM1_ESM.docx]

**Appendix A**

**Table 1.** Observation depth of deformation and groundwater level at the WSY station

| Extensometers | Observation layer | Lithology | Water-level  observation well | Observation layer | Lithology |
| --- | --- | --- | --- | --- | --- |
| F1-7 | 2-15.2 | Silt/medium-fine sand | D1-5 | 9.2-14.7 | Medium-fine sand |
| F1-6 | 15.2-24 | Silty clay |  |  |  |
| F1-5 | 24-48 | Medium-coarse sand/clay | D1-4 | 24.8-46.3 | Medium-coarse sand |
| F1-4 | 48-65.9 | clay |  |  |  |
| F1-3 | 65.9-94 | Medium-coarse sand/clay | D1-3 | 64.9-94 | Medium-fine sand  containing gravel |
| F1-2 | 94-147.6 | Silt/fine sand | D1-2 | 124.1-146.7 | Fine sand/gravel |
| F1-1 | 147.6-182 | Clay/medium-fine sand | D1-1 | 159.7-182.8 | Medium-fine sand  / gravel |

**Table 2.** Observation depth of deformation and groundwater level at the WJ station

| Extensometers | Observation layer | Lithology | Water-level  observation well | Observation layer | Lithology |
| --- | --- | --- | --- | --- | --- |
| F2-7 | 2.2-18.4 | Silt/fine sand | D2-5 | 9.2-16.02 | Fine sand |
| F2-6 | 18.4-29 | Silty clay |  |  |  |
| F2-5 | 29-64.5 | Silty clay/medium-fine sand | D2-4 | 39.36-63.68 | Medium sand |
| F2-4 | 64.5-80 | Clay |  |  |  |
| F2-3 | 80-99 | Silty clay/medium-fine sand | D2-3 | 79.86-96.88 | Medium-fine sand |
| F2-2 | 99-133 | Medium-coarse sand/clay | D2-2 | 119.78-132.16 | Medium-coarse sand/sand gravel |
| F2-1 | 133-199 | Medium-coarse sand/silt | D2-1 | 180.03-198.66 | Medium sand/sand gravel |

**Table 3.** Observation depth of deformation and groundwater level at the TZ station

| Extensometers | Observation layer | Lithology | Water-level  observation well | Observation layer | Lithology |
| --- | --- | --- | --- | --- | --- |
| F3-10 | 2.4-35.4 | Silt | D3-6 | 27.5-31.0 | Silt |
| F3-9 | 35.4-48.5 | Clay |  |  |  |
| F3-8 | 48.5-64.5 | Fine sand/coarse sand | D3-5 | 59.3-63.4 | Fine sand/medium  coarse sand with gravel |
| F3-7 | 64.5-82.3 | Clay/silty clay |  |  |  |
| F3-6 | 82.3-102 | Silty clay/fine sand | D3-4 | 85.7-91.3 | Fine sand |
| F3-5 | 102-117 | silty clay |  |  |  |
| F3-4 | 117-148.5 | Silt/coarse-fine sand | D3-3 | 120.0-146.8 | Coarse-fine sand |
| F3-3 | 148.5-218.9 | Silt/fine sand | D3-2 | 210.0-218.0 | Coarse sand  containing gravel |
| F3-2 | 218.9-238.1 | Silty clay |  |  |  |
| F3-1 | 238.1-308 | Silt/sand | D3-1 | 245.0-308.0 | Medium-coarse  sand |

**Appendix B**

**Table 1.** Parameters of the change points detection result of D1-3

| **Time** | **Confidence Interval** | **Confidence level** | **Level** |
| --- | --- | --- | --- |
| Apr, 2005 | Apr, 2005 ~ Jul, 2005 | 98% | 2 |
| Mar, 2007 | Mar, 2007 ~ Mar, 2007 | 100% | 1 |
| Mar, 2009 | Sep, 2008 ~ Mar, 2009 | 98% | 2 |

**Table 2.** Parameters of the change points detection result of F1-3

| **Time** | **Confidence Interval** | **Confidence level** | **Level** |
| --- | --- | --- | --- |
| May, 2005 | May, 2005 ~ May, 2005 | 100% | 3 |
| Jun, 2007 | Jun, 2007 ~ Jun, 2007 | 94% | 1 |
| Apr, 2008 | Apr, 2008 ~ Apr, 2008 | 93% | 3 |
| Dec, 2009 | Dec, 2009 ~ Dec, 2009 | 97% | 3 |

**Table 3.** Parameters of the change points detection result of D2-3

| **Time** | **Confidence Interval** | **Confidence level** | **Level** |
| --- | --- | --- | --- |
| Oct, 2005 | Oct, 2005 ~ Jan, 2006 | 100% | 1 |
| Sep, 2008 | Jun, 2008 ~ Sep, 2008 | 100% | 2 |

**Table 4.** Parameters of the change points detection result of F2-3

| **Time** | **Confidence Interval** | **Confidence level** | **Level** |
| --- | --- | --- | --- |
| Oct, 2005 | Oct, 2005 ~ Oct, 2005 | 100% | 3 |
| Mar, 2007 | Mar, 2007 ~ Mar, 2007 | 99% | 2 |
| Oct, 2008 | Sep, 2008 ~ Dec, 2008 | 100% | 3 |

**Table 5.** Parameters of the change points detection result of D3-4

| **Time** | **Confidence Interval** | **Confidence level** | **Level** |
| --- | --- | --- | --- |
| Apr, 2007 | Oct, 2006 ~ Oct, 2007 | 100% | 1 |

**Table 6.** Parameters of the change points detection result of F3-7

| **Time** | **Confidence Interval** | **Confidence level** | **Level** |
| --- | --- | --- | --- |
| Oct, 2005 | Oct, 2005 ~ Oct, 2005 | 99% | 3 |
| Jul, 2007 | Jul, 2007 ~ Jul, 2007 | 99% | 4 |
| Apr, 2009 | Apr, 2009 ~ Apr, 2009 | 99% | 2 |
